# Intrinsic Brain Connectivity in Chronic Pain: A Resting-State fMRI Study in Patients with Rheumatoid Arthritis

**DOI:** 10.3389/fnhum.2016.00107

**Published:** 2016-03-15

**Authors:** Pär Flodin, Sofia Martinsen, Reem Altawil, Eva Waldheim, Jon Lampa, Eva Kosek, Peter Fransson

**Affiliations:** ^1^Department of Clinical Neuroscience, Karolinska InstitutetStockholm, Sweden; ^2^Department of Medicine, Rheumatology Unit, CMM, Karolinska Institutet, Karolinska University HospitalStockholm, Sweden

**Keywords:** rheumatoid arthritis, pain, inflammation, joint, fMRI, resting-state, brain connectivity

## Abstract

**Background:** Rheumatoid arthritis (RA) is commonly accompanied by pain that is discordant with the degree of peripheral pathology. Very little is known about the cerebral processes involved in pain processing in RA. Here we investigated resting-state brain connectivity associated with prolonged pain in RA.

**Methods:** 24 RA subjects and 19 matched controls were compared with regard to both behavioral measures of pain perception and resting-resting state fMRI data acquired subsequently to fMRI sessions involving pain stimuli. The resting-state fMRI brain connectivity was investigated using 159 seed regions located in cardinal pain processing brain regions. Additional principal component based multivariate pattern analysis of the whole brain connectivity pattern was carried out in a data driven analysis to localize group differences in functional connectivity.

**Results:** When RA patients were compared to controls, we observed significantly lower pain resilience for pressure on the affected finger joints (i.e., P50-joint) and an overall heightened level of perceived global pain in RA patients. Relative to controls, RA patients displayed increased brain connectivity predominately for the supplementary motor areas, mid-cingulate cortex, and the primary sensorimotor cortex. Additionally, we observed an increase in brain connectivity between the insula and prefrontal cortex as well as between anterior cingulate cortex and occipital areas for RA patients. None of the group differences in brain connectivity were significantly correlated with behavioral parameters.

**Conclusion:** Our study provides experimental evidence of increased connectivity between frontal midline regions that are implicated in affective pain processing and bilateral sensorimotor regions in RA patients.

## Introduction

Rheumatoid arthritis (RA) is a chronic, autoimmune inflammatory disease that primarily affects the joints. The prevalence of RA is estimated to be 0.5–1% of the population in the industrialized world, with an overrepresentation of women (McInnes and O'Dell, 2010). The inflammation may lead to dysfunction and destruction of joints, accompanied with joint pain.

Pain severely impacts the patients' perceived subjective health. However, there are often large discrepancies between objective RA inflammatory markers and the degree of subjective pain (Thompson and Carr, 1997). Similarly, although a multitude of efficient immunosuppressive and biologic therapies have proven efficient for a majority of the RA patients, many patients continue to experience significant pain despite improvements in peripheral joint inflammation (Taylor et al., 2010). It is thus reasonable to stipulate that the long-term pain in RA may be accompanied by altered cerebral pain processing, which is also indirectly supported by previous studies showing a generalized increase in pain sensitivity in RA patients compared to controls (Leffler et al., 2002; Fridén et al., 2013). Increased knowledge of the cerebral response to prolonged rheumatic pain could thus be valuable for the development of pharmacological and behavioral therapies aimed at reducing pain in RA. In line with our previous studies of altered resting-state connectivity (Flodin et al., 2014) and abnormal cerebral pain processing in fibromyalgia patients (Jensen et al., 2009), a limited number of studies have investigated pain processing in RA populations. For instance, Wartolowska et al. (2012) used structural MR imaging and reported that RA patient (vs. HC) displayed increased gray matter density in the basal ganglia which is involved in motor control and pain processing. Other studies have targeted brain activation patterns evoked by pain. Jones and Derbyshire (1997) reported reduced brain response to heat induced pain in prefrontal regions and the anterior cingulate cortex. Schweinhardt et al. (2008) on the other hand, found correlations between pain evoked brain activity in the medial prefrontal cortex (MPFC) and depressive symptoms in RA patients. Thus, there are corroborating results from different imaging modalities that RA is associated with an altered state of central pain processing, which likely could be ascribed to the prolonged pain experience. However, to our knowledge, the current study is among the first to investigate spontaneous fluctuation of brain activity in canonical pain brain regions among RA patients using resting state fMRI.

Our main hypothesis was that long-term pain that accompanies RA would influence intrinsic brain connectivity of pain relevant regions. Furthermore, we hypothesized that the intensity of RA related pain and pain sensitivity (e.g., ratings of global pain intensity and pressure sensitivity of affected joints) would correlate with the presumptive group differences in functional connectivity.

## Methods

### Subjects

Rheumatoid arthritis patients were recruited through the rheumatology clinic at the Karolinska Hospital in Stockholm, Sweden. Patients fulfilling the inclusion criteria were asked to participate in a randomized, placebo-controlled trial investigating the effects of a tumor necrosis factor (TNF-alpha) blocker on pain and inflammation in RA with baseline comparison with healthy subjects (the PARADE study; www.clinicaltrials.gov; identifier NCT01197144, EudraCT 2009-017163-42).

In this report, only results from the baseline data are described. Inclusion criteria for the RA patients were working age (≥18 years), meeting the ACR 1987 classification criteria for RA (Arnett et al., 1988), clinical indication for use of TNF-blockers and MR examination compatibility. Exclusion criteria were left handedness, fibromyalgia, severe cardiovascular disease, vasculitis, neurological disease, ongoing treatments for anxiety or depression using antidepressants and other reasons based on the judgment of the responsible physician.

For the age- and sex matched healthy controls, exclusion criteria were identical to the RA patients with the additional exclusion criteria of recurrent pain problems, including RA and fibromyalgia.

In total, 27 RA patients were recruited for participation in the study. Two patients were discarded due to excessive head movement during resting state fMRI scanning. Movement outlier participants were identified using mean frame wise displacement (FD) >0.31 mm, corresponding to two standard deviations from the mean of all subjects. Data from one subject had to be rejected due to partial head coverage, rendering 24 RA subjects to be eligible for inclusion in the analysis. Mean age was 53.8 years (range 23–74 years), and 20 were females. Among the 24 RA patients, 17 used Methotrexate, 3 Sulphasalazine and 1 Leflunomide. No patient used higher cortisone dose than 10 mg. See Supplemental Table S1 for individual medication usage and Table 1 for further population characteristics.

**Table 1:** **Data cohort characteristics**.

	**RA (*n* = 24)**	**HC (*n* = 19)**
Age (mean ± SD)	53.8 ± 14.8	50.4 ± 16.6
Gender (F/M)	20/4	16/3
FD (mean ± SD)	0.15 ± 0.068	0.11 ± 0.036
P50 thumb (mean ± SD)	584.2 ± 186.5	608.7 ± 181.5
P50 joint (mean ± SD)	505.7 ± (262.8)	758.4 ± 126.0
Global Vas (mean ± SD)	33.7 ± (29.3)	0.95 ± 3.44
DAS28 (mean ± SD)	5.20 ± (1.14)	–
RA duration (m) (mean ± SD)	66.0 ± (34.0)	–
Swollen joints (mean ± SD)	7.25 ± (5.06)	–
Tender joints (mean ± SD)	9.79 ± (6.35)	–

Twenty-one healthy age- and sex matched control subjects (HC) were recruited through advertisements on noticeboard primarily at the hospital campus. fMRI data from two subjects were discarded due to excessive head movement, leaving 19 HC for further analysis (mean age 50.42 years, range 25–68 years, 16 females).

Screening of RA subjects was performed at the first visit to the hospital. During the first visit, all subject's sensitivity to evoked pressure (P50) was calibrated. Subjects returned the following day for the fMRI scanning.

The study conforms with Swedish legislation regarding clinical pharmacological trials and necessary permit from the Swedish medical products agency has been obtained. The regional ethics committee in Stockholm approved the study and informed consent was obtained from all participants.

### Clinical and behavioral measures

#### Assessment of pressure pain sensitivity

To assess pain sensitivity, we applied an automatic, pneumatic computer-controlled stimulator with a plastic piston corresponding to an area of 1 cm^2^ (Jensen et al., 2009) on the thumbnail and on the most affected finger joint (or the corresponding joint in healthy controls). Subjects rated the pain intensity of the pressure stimuli on a visual analog scale (VAS). For both locations we first used ascending stimuli to determine the pressure pain threshold and the first pressure rated as >60 mm on VAS. Each subject was then stimulated with five pressure intensities evenly distributed within this interval, 3 times for each intensity, in a randomized order. The stimuli were presented for 2.5 s with a 30 s inter-stimulus interval. A linear polynomial function was fitted to the 15 data points, and from this we derived a measure of 50 mm VAS, that we referred to as P50 (for further details, see Jensen et al., 2009).

#### Assessment of global pain

Prior to the fMRI scan subjects were asked to rate their overall pain intensity using a 100 mm VAS, spanning from “no pain” to “worst imaginable pain” (here referred to as VAS global pain).

#### RA disease activity

For estimating RA activity, we calculated the Disease Activity Score, DAS28, (Prevoo et al., 1995). DAS28 is a composite measure of the number of tender joints in 28 locations, the number of swollen joints, patients' perceived global health and an inflammatory marker of erythrocyte sedimentation rate (ESR). DAS28 was determined on the day before the fMRI scanning.

### MRI data acquisition

MR imaging was performed on a 3T General Electric 750 MR scanner installed at the MR Research Center, Karolinska Institute, Stockholm. Anatomical MR imaging was acquired with a high-resolution BRAVO 3D T1-weighted image sequence (1 × 1 × 1 mm3 voxel size). For each subject we performed one resting state scan consisting of 200 volumes, using an echo-planar imaging with TR/TE = 2500/30 ms, flip angle = 90°, 49 slices, 96 × 96 matrix size, FOV = 288 × 288 mm, slice thickness = 3 mm and an interleaved mode of slice acquisition. Anatomical (T2-weighted) scans were investigated by radiologist for clinical abnormalities. In the resting state condition, subjects were instructed to lie still and rest, and not to think of anything in particular while keeping their eyes on a fixation cross. Prior to the resting state fMRI data acquisition, subjects underwent two fMRI sessions of a pain exposure paradigms (~7 min each). Results from the task-evoked fMRI runs will be reported elsewhere.

### Resting state fMRI data analysis

Group differences in resting state activity, as well as strength of functional connectivity correlating with clinical and behavioral pain measures within the RA group were investigated using a seed-based correlation analysis (SCA). Seed selection was based on 159 uniformly placed spherical ROIs (4 mm radius, 10 mm apart center-to-center) within brain regions that are known to be involved in pain processing. Brain regions related to pain was demarked in a meta-analysis of 314 pain studies indexed in neurosynth (neurosynth.org, retrieved in December 2013), identical to the set of ROIs described in Flodin et al. (2014). This seed selection procedure aimed to enhance sensitivity by restricting SCA to only pain relevant seeds rather than a set of seeds that cover the whole brain. Thereby, we decrease the magnitude of the multiple comparison problem, while at the same time allowing for an extensive set of seed to be used that lessen the influence of seed selection bias. The seed region coordinates and associated anatomical labels are listed in Supplemental Table S2 and shown in Supplemental Figure S1.

Prior to SCA, imaging data were preprocessed using SPM8 (Welcome Trust Center of Neuroimaging, University College London, UK). Image preprocessing included slice-time correction, realignment to the mean image, co-registration of functional and structural images, tissue segmentation of structural images, and direct normalization of functional and structural scans to the MNI template provided by SPM8. Finally, functional volumes were spatially smoothed using an 8 mm FWHM Gaussian kernel. Subject level SCA analyses were carried out using the Conn toolbox (http://www.nitrc.org/projects/conn; Whitfield-Gabrieli and Nieto-Castanon, 2012). Functional volumes were band pass filtered at 0.008–0.09 Hz (default values) simultaneously with nuisance regression (as advocated by Hallquist et al., 2013, in order not to reintroduce nuisance -related variations into a band-pass filtered time-series). Subject specific nuisance regressors included 6 movement regressors and their time derivatives, and 5 regressors pertaining to white matter and CSF signals sources respectively, using a principal component (PCA) based noise correction (CompCorr) approach (Behzadi et al., 2007). Additionally, images that were regarded as movement outliers were regressed out. Image volume outliers were detected using the ART toolbox (nitrc.org/projects/artifact_detect/) and defined as image volumes with a frame wise displacement (FD) value larger than 0.5 mm or signal intensity changes greater than 3 standard deviations (default thresholds). Outlier volumes were modeled at the first level general linear model using dummy variables and regressed out together with the other subject specific nuisance regressors. The mean number of regressed volumes for RA subjects was 13.3 ± 12.5 *SD*, and 5.6 ± 5.4 *SD* among HC. Across the cohort, the number of regressed volumes ranged between 0 and 46 volumes. Thus, all subjects had an equivalent of at least 6 min 25 s of resting state scans (i.e., 77% of the original data points were not regressed out). There was a significant group difference with regard to number of scrubbed volumes [*t*_(41)_ = 2.51, *p* = 0.016].

For each subject and each seed region, z-transformed Pearson correlation maps were used in the second level group analyses. All second level group analyses were controlled for mean FD, age and sex. Independent *t*-tests were used for testing group differences in functional connectivity for each seed region. Furthermore, using measures of pain sensitivity we investigated how pain sensitivity affected functional connectivity across subjects in both groups. All reported SCA results are thresholded at a false discovery rate (FDR) corrected cluster level of *p* < 0.05/159 = 0.00031, accounting for 159 *t*-tests using Bonferroni correction. Cluster defining voxel threshold was *p* < 0.001, uncorrected (to minimize number of false positive and negatives, see Woo et al., 2014).

Additionally, we performed a principal component based multivariate pattern analysis (MVPA) to detect group differences with regard to whole brain connectivity for each voxel. The MVPA analysis complements the SCA in that it is not limited to investigating functional connectivity in pre-selected pain regions, but provides a regionally unbiased mapping of brain areas with abnormal whole brain connectivity patterns. Whereas a SCA conducted on seeds defined in each gray matter voxel requires a very conservative multiple comparison correction that likely would prevent any significant group differences, the MVPA approach enabled us to detect putative abnormal connectivity patterns at a whole brain level using one simple F-test. In detail, the MVPA measure was obtained by reducing each voxels whole brain connectivity matrix into three principal components. The whole brain connectivity matrix for each voxel was reshaped into a row vector and subsequently concatenated over all participants into a matrix NxV, where N was the number of subjects and V is the number of voxels within the brain mask. The dimensionality of the NxV group correlation matrix was reduced by principal component analysis (PCA). This yielded an NxC matrix, where C is the number of maintained principal components. We maintained the first three principal components that explained the most of the variance of the connectivity matrix (*C* = 3), resulting in three component score volumes that best represented the whole brain connectivity pattern for each subject. These volumes were included in an *F*-test on the group level. Thus, we tested for clusters that differed between RA patients and HC with regard to whole brain connectivity as represented by the PCA component volumes. Subsequently, we performed a *post-hoc* seed correlation analysis, using spherical seeds placed at the peak voxels at the three clusters from the MVPA (MNI coordinates x, y, z: −28, 42, 58; 22, −14, 56; 10, 46, 44). The purpose of this analysis was to further probe the nature of putative group related differences in connectivity patterns of these regions (**Figure 2**). All group analyses were controlled for mean frame wise displacement, sex, and age.

## Results

### Behavior

RA patients rated higher levels of overall pain (VAS global pain); *t*_(41)_ = 4.84, *p* < 0.00001 than HC. We observed significantly increased pressure pain sensitivity at the affected finger joints (i.e., P50 joint) in RA patients compared to controls *t*_(41)_ = −3.85, *p* = 0.0002. However, the groups did not differ in pain sensitivity at the thumbnail (P50 thumb), *t*_(41)_ = 0.43, *p* = 0.69.

### Functional brain connectivity

**Figure 1:**
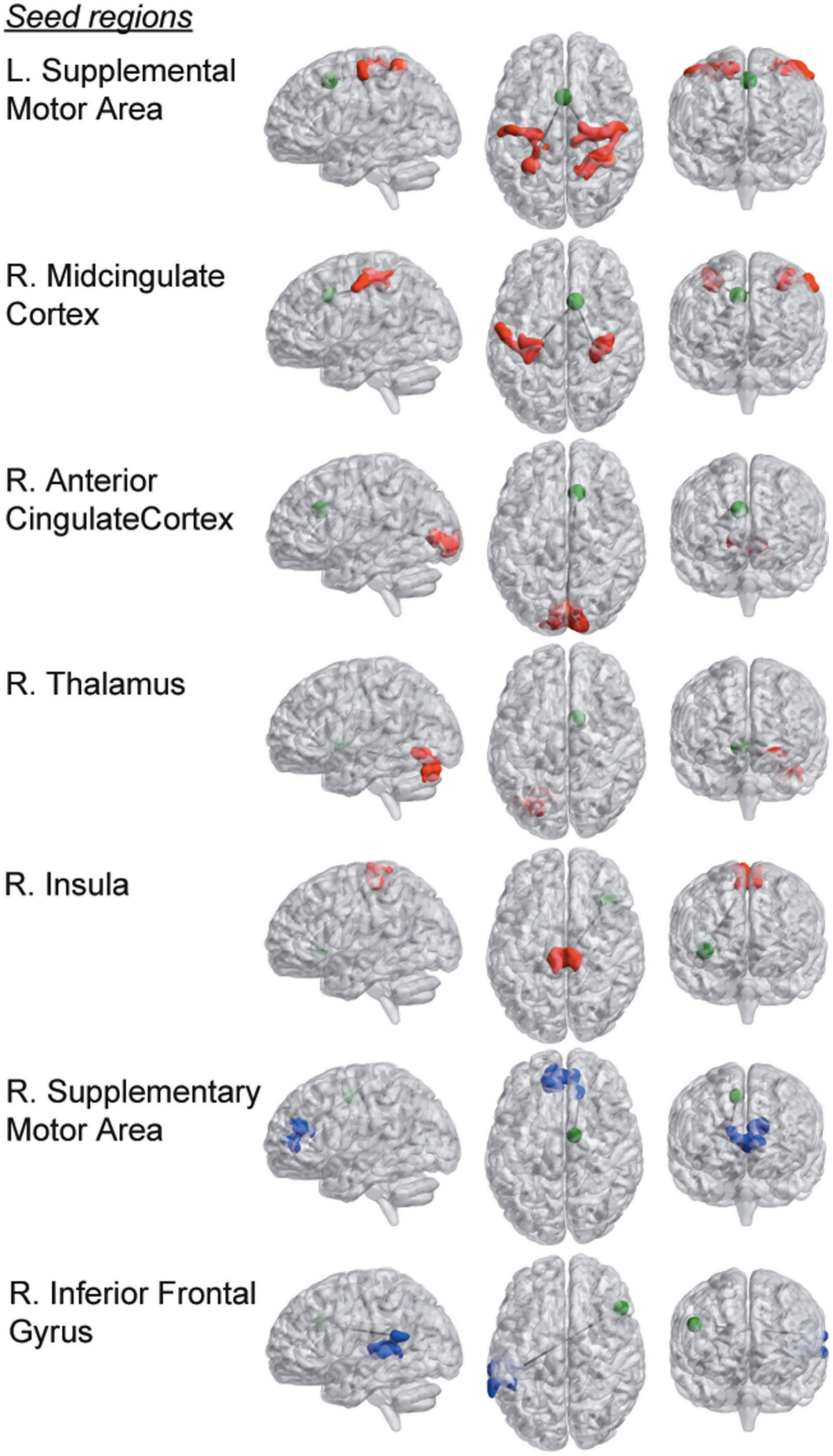
**Group differences in functional connectivity of the 159 a-priori defined seed regions within pain processing brain areas**. Seed regions are depicted as green spheres (the radius of the seed points has been increased by a factor of 2 for display purposes). Brain areas that display stronger connectivity with respective seed region in the RA compared to the HC group are colored in red, and blue areas represent clusters that are more strongly connected in the HC compared to the RA cohort (*p* < 0.0031, FDR-corrected at the cluster level).

Furthermore, we observed seven group differences with regard to functional connectivity of the original 159 pain regions that were investigated. Overall, the observed pattern of connectivity differences in RA compared to HC was an increase in connectivity between tested pain seeds and other parts of the brain (see Figure 1, Table 2). Most prominently, RA patients displayed an elevated level of connectivity for seed regions located in both the supplementary motor area and in the mid-cingulate cortex with bilateral primary sensory motor cortices. In addition, we observed an increased level of connectivity for RA between the insula and premotor regions. We also observed an unexpected increased occipital connectivity (to thalamus and ACC) in the RA cohort.

**Table 2:** **Group differences in functional connectivity**.

**Contrast**	**Seed (center of sphere)**	**Target(s) (peak coordinates)**	**Clustersize (# voxels)**	**Cluster p-FWE**
RA>HC	Supplementary Motor Area (0, 14,48)	S1/M1 (39, −42, 54)	1603	<0.000001
		S1M1 (−30, −48, 60)	769	0.000078
	Med. Front. Sup. Gyr. ^a^ (10, 46, 44)	Somatosensory (6, −70, 42)	1579	<0.000001
		Premotor (38, 16, 42)	708	0.00010
	ACC (10, 24, 28)	SVC (0, −82, 0)	1462	<0.000001
	Insula (40, 24, −2)	Premotor (2, −28, 60)	931	0.000025
	MCC (10, 14, 38)	S1/M1 (−30, −32, 50)	797	0.000062
		S1/M1 (30, −32, 50)	665	0.00025
	Thalamus (10, 4, −2)	AVC (−24, −76, −18)	705	0.00013
HC>RA	Postcentral Gyrus ^a^ (−28, −42, 58)	AVC (34, −56, −10)	822	0.000033
	Supplementary Motor Area (10, −6, 48)	dACC (16, 34, 18)	816	0.000037
	Inf. Frontal Gyrus (50, 24, 28)	Supplementary Temporal Gyrus (−64, −44, 18)	810	0.000066

*Target regions are labeled based on the locations of the largest number of voxels within significant cluster, as identified and labeled within the CONN-toolbox. SMA, supplementary motor area; S1/M1, primary sensorimotor regions; MCC, middle cingulate cortex; SVC, secondary visual cortex; (d) ACC, (dorsal) anterior cingulate cortex; AVC, associative visual cortex*.

a *Seed regions defined post-hoc based on results from the MVPA analysis. All results are significant on a corrected cluster level (p < 0.00031, FDR), Bonferroni corrected for 159 seed correlation analyses (SCA)*.

For two a priori seeds regions, we detected weaker connectivity in the RA. The functional connectivity between supplementary motor area (SMA) and dorsal anterior cingulate cortex (dACC), and between inferior frontal gyrus and superior temporal gyrus were both lower in RA compared to HC. (Parametric T-maps for all group differences listed in Table 2 are available in NeuroVault at: http://neurovault.org/collections/1151).

**Figure 2:**
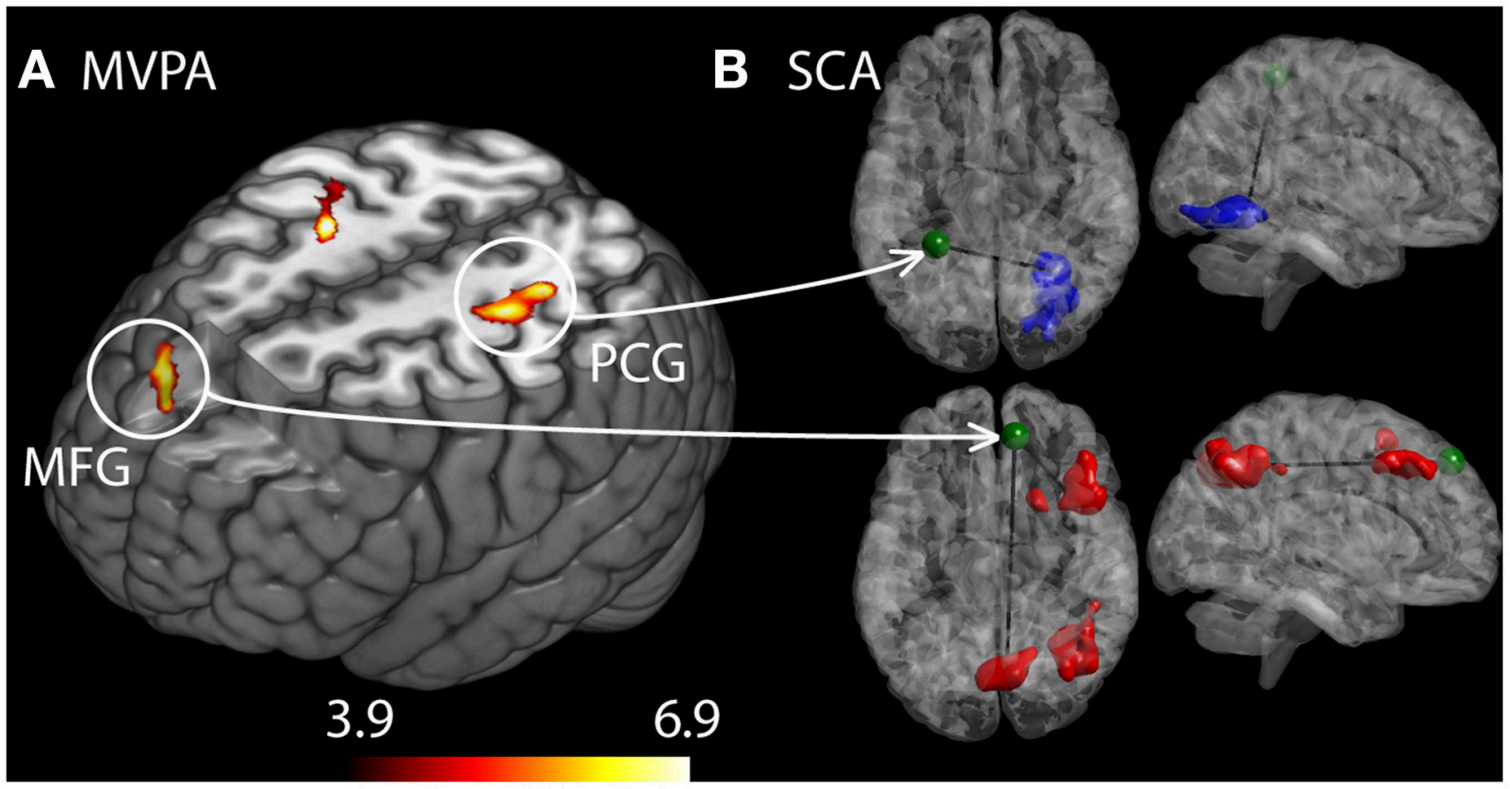
**Differences in brain connectivity profile indexed at a voxel level using multivariate pattern analysis (MVPA). (A)** Brain regions that are significantly different between the RA and the HC cohorts with regard to connectivity profiles. Colorbar indicate F-statistic of between group differences with regard to the spatial maps of the three first principal components. Three clusters were identified: in left middle frontal gyrus (MFG) and in left and right post central gyrus (PCG). The F-maps are threshold at a *p* < 0.05 FDR corrected cluster level, using an explorative voxel level threshold of *p* < 0.01. **(B)** *Post-hoc* SCA using seed regions defined as spheres placed at the peak coordinates from MVPA, identified RA increased functional connectivity between MFG and premotor areas as well as a cluster spanning the precuneus and somatosensory areas (red clusters). HC displayed stronger connectivity between left PCG and contralateral associative visual areas.

The MVPA analysis showed group differences with regard to whole brain functional connectivity patterns in three regions, located in medial frontal gyrus (MFG) and bilateral somatosensory cortex in post central gyrus (PCG) (see Figure 2A). In the *post-hoc* seed correlation analyses, where we conducted SCA using seed regions based on the significant group differences in the MVPA, we found stronger connectivity between medial frontal gyrus (MFG) and premotor and somatosensory regions for HC, as well as decreased connectivity between post central gyrus (PCG) and associative visual cortex (ASV) in RA patients relative HC.

None of the observed group differences in connectivity correlated with any of the measures (listed in Table 1, i.e., P50 thumb, P50 joint, global VAS, DAS28 or RA duration), after Bonferroni correction for multiple comparisons (9 group differences in connectivity rendered a Bonferroni corrected *p*-value of 0.05/9 = 0.0056). Associations between clinical symptoms and functional connectivity (controlled for sex, age, and FD) was quantified using Pearson correlation statistic within the RA group. However, in the RA cohort there was a trend [here defined as *p*-values above the bonferroni corrected *p*-value, (i.e., 0.0056 < 0.05)] for a negative correlation between DAS28 scores and the connectivity between SMA and S1/M1, as well as a trend of a positive correlation between DAS28 scores and the connectivity between the inferior frontal gyrus (IFG) and premotor- and sensorimotor areas (*r* = 0.44, *p* = 0.033). Furthermore, there was a trend of a positive correlation between pain sensitivity on thumb (e.g., P50 thumb) and the strength of connectivity between right supplementary motor area and dACC (*r* = 0.46, *p* = 0.021).

Since we observed stronger movement in the RA group compared to the HC, we performed a *post-hoc* control analysis to investigate whether the connectivity differences were related to head movement (i.e., mean framewise displacement). Despite the rigorous set of strategies employed to minimize the effect of head motion in the group comparisons (see the Method section), we did find a significant relationship between head-movement (framewise displacement) and connectivity within the RA group. There was a negative correlation between mean framewise displacement and the SMA-S1/M1 connectivity (*r* = −0.58, *p* = 0.0030), and between the thalamus-AVC connectivity and movement (*r* = −0.65, *p* = 0.00058). We also found a trend for a negative relationship between movement and ACC connectivity (*r* = −0.43, *p* = 0.036), and a positive relationship between movement and the connectivity between inferior frontal gyrus (IFG) and superior temporal gyrus (STG; *r* = 0.40, *p* = 0.050).

## Discussion

In the present study we could confirm that RA patients suffer from increased sensitivity to supra-threshold pressure pain (i.e., hyperalgesia) in affected joints, but we found no signs of generalized hyperalgesia. The fact that hyperalgesia was confined to the affected joints in current study is in accordance with peripheral sensitization due to ongoing inflammation, but does not support a more generalized increase in pain sensitivity in our RA patients. The discrepancy between the present study and previous studies could depend on different methodologies (i.e., method of levels vs. method of limits in the previous studies) or on differences in patient cohorts. In addition, as expected, RA patients rated a higher global pain intensity compared to controls.

With regard to functional connectivity, we observed several important abnormalities. The main results are increased connectivity between frontal midline regions [the seeds in SMA, middle cingulate cortex (MCC) and middle frontal gyrus (MFG)] implicated in affective pain processing, to bilateral sensorimotor regions in RA. However, none of the tested group differences in connectivity correlated with measures of symptom gravity within the RA group after Bonferroni correcting for multiple tests. This prevents any firm conclusions on the functional significance of the group differences in functional connectivity to be made. Although the functional interpretation of increased frontal-S1/M1 connectivity remains to be established directly, one could speculate that it reflects increased attribution of emotional valiance to pain stimuli for the cohort of RA patients. Support for this interpretation is obtained from studies on back pain that reports a gradual shift of cerebral pain representations from canonical nociceptive regions toward affective circuits (although non-overlapping with the circuits reported here; Baliki et al., 2012; Hashmi et al., 2013). An alternative but not mutually exclusive interpretation is that the observed hyper-connectivity reflects an increased demand and taxation of prefrontal top-down regulation of sensory areas. For instance (Jones and Derbyshire, 1997) showed a decreased cerebral response in ACC and other prefrontal regions in ACC for induced pain among RA (*n* = 6) patients. The diminished prefrontal pain response among RA patients was interpreted as reflecting an adaptive cognitive and psychological response. A third option is that the altered sensorimotor connectivity is a consequence of life style changes in motor behavior due to a prolonged exposure to pain in the RA cohort. Unfortunately, we did not collect detailed data on motor habits, thus preventing us to test such relationships. It should be noted that the discussion above rely on reverse inference, that is, an inference of the functional significance based on what is known from earlier studies about the functional role of these areas. Since any brain region typically is involved in multiple cognitive processes, such inference is severely limited (for an in depth discussion, see e.g., Poldrack, 2011). Further investigation using complementary measures of for example the degree of daily activity (such as pedometers) would be interesting for determining whether group differences are related to altered movement patterns or other factors besides the exposure to chronic rheumatic pain.

Altered functional connectivity in RA patients shows that prolonged nociception, either alone or in combination with other lifestyle changes associated with RA (e.g., changes in physical activity or mood), modulates the brain connectivity pattern in resting state fMRI. Modulation of resting state brain connectivity in response to behavior and external factors is an intriguing phenomenon that have been confirmed in a wide range of contexts, including cognitive training, physical exercise and motor practice (for a review, see Guerra-Carrillo et al., 2014). Brain imaging of chronic pain patients have identified abnormal pain processing, such as deficiency in inhibitory pain circuits (Jensen et al., 2012), and abnormal resting state brain activity in fibromyalgia patients (Napadow et al., 2012; Flodin et al., 2014). The nature of abnormal cerebral pain processing in chronic pain conditions is far from established, and the heterogeneity of results between different chronic pain studies are likely due to differences in the cohorts investigated, the kind of tasks or no tasks used, and the analytical approaches employed to analyze the MR data. However, a general finding of the neuroimaging literature on rheumatic pain is the central role of the (medial) pain system for sensitization and pain inhibition, according to a review by Jones et al. (2012). They further proposed that studying the brains baseline activity (i.e., resting state fMRI) likely would prove fruitful for increasing our understanding of these mechanisms. Recently we investigated the resting state brain activity in FM by employing similar approaches for fMRI acquisition and data analysis as for the current study (Flodin et al., 2014). The main finding consisted in a reduced connectivity between pain and sensorimotor regions in fibromyalgia (FM). Similarly, Pujol et al. (2014) investigated resting state functional connectivity in FM and concluded that FM displays a general weakening of sensory integration, which could underlie the clinical pain in FM. However, the arguably most recurrent finding with regard to resting state activity in centralized pain is increased connectivity between insula and DMN (Napadow and Harris, 2014). Although we failed to replicate increased insula-DMN connectivity in FM, we observed a significant correlation between pain sensitivity (i.e., inverted P50 thumb) and the functional connectivity between insula and posterior cingulate cortex in the DMN. In contrast, the association of the insula-DMN connectivity and the degree of pain sensitivity was not replicated in the current study. The replication failure could be due to differences in patient cohorts, or other factors contributing to the inherent noise of the both the fMRI measurements and behavioral estimates of pain sensitivity (Barch and Yarkoni, 2013).

In a commendable attempt, Sundermann et al. (2014) used support vector machines to classify resting state brain activity in RA relative FM based on the connectivity within and between nodes of the DMN and the salience network. However, neither the conventional univariate analytical approach, nor the multivariate approaches of support vector machines rendered significant group differences. Interestingly however, the same research group previously identified a pattern of mostly opposing pain evoked brain activation for RA compared to FM. These were located in prefrontal regions and thalamus (Burgmer et al., 2009). Thus, it is worth comparing the current characterization of resting state connectivity in RA that mainly consisted of increased connectivity between pain-and sensorimotor regions, with the opposing picture that pain in FM is associated with sensory disintegration (Flodin et al., 2014; Pujol et al., 2014). Speculatively, these connectivity differences could relate to the proposal that RA, perhaps in contrast to FM, partly involves an adaptation that serves to decrease the experienced pain (Jones and Derbyshire, 1997).

However, the RA group also displayed decreased connectivity. Importantly, the decreased connectivity between SMA and dACC observed in RA pertains to a part of the supplementary motor area that frequently has been associated with noxious perception (Duerden and Albanese, 2013). The target region in ACC (see Figure 1 and Table 2) is located in the vicinity to the brain area that we previously showed to be under-recruited by chronic pain patients (fibromyalgia) in response to evoked pain. This hypo-connectivity was interpreted as a deficient top-down control of descending pain pathways (Jensen et al., 2009). Furthermore, here we observed a tendency of positive correlation between P50 joint (e.g., pain resilience) with SMA—dACC connectivity (*r* = 0.46, *p* = 0.021) in the RA cohort. Thus, a failure to recruit prefrontal control networks could be present in individual suffering from either of the two rheumatic pain conditions.

A limitation and possible confound in the reported results is the fact that the RA subjects were more prone to move during resting state scanning compared to HC. Typically, micro-head movement is associated with decreased long range anterior-posterior connectivity, and increased bilateral short range connectivity between the hemispheres (Power et al., 2012). Although we have undertaken a set of proven strategies modeling (scrubbing, inclusion of nuisance regressors at the first level and mean FD values at the second level of analysis) to counteract the effect of group differences in movement, there still remained correlations between movement and connectivity. However, matching the groups with regard to movement would have biased the RA group toward a less representative RA cohort. Since the direction of the correlations between movement and connectivity was negative (that is, more movement was associated with less connectivity), movement or the rigorous approach to movement correction likely decreased rather than induce the observed group differences. Movement was, or tended to be related to several of the group differences in connectivity involving occipital regions. For instance, the thalamic- AVC connectivity is normally very weak or absent (as verified in the sample of 1000 subjects available at neurosynth.org), and the enhanced FC between these regions are difficult to interpret in disease relevant terms. Similarly, the observed increased ACC-SCA connectivity in RA had a tendency of correlation with movement, and could partly be confounded by movement.

A second limitation and possible confound in the current study is the fact that resting state scans were acquired subsequent to task-based pain fMRI sessions (that will be reported elsewhere), possibly introducing spill-over effects into the resting-state data (Stevens et al., 2010; Wang et al., 2012). For instance, one could imagine that chronic pain patients experience stronger after-effects from a pain paradigm than healthy controls do, and that such asymmetry would confound group comparisons of the intrinsic brain activity.

Future studies would benefit from larger cohort sizes. In addition to the conventional beneficial effects of greater sample sizes on statistical sensitivity, a larger RA cohort would allow for subdivision of the cohort based on affected joint. Using functionally localizers, one could examine the connectivity of the cortical regions that are involved in processing pain of the primarily affected joints. Additional improvement in the seed selection by using functional localizers could narrow down the number of seeds tested, thus lessen the conservative impact of Bonferroni correction.

## Conclusion

In the current study we have examined how RA patients differ from HC with regard to resting state functional connectivity. The general pattern that emerged was a stronger connectivity of regions in the medial pain system and regions in sensory- and motor cortex in RA. Additionally, RA related hypo- connectivity was found between frontal control areas and premotor regions that are associated with processing of noxious stimuli. However, the functional role of the group differences in connectivity remains to be established since the associations to subjective pain data and clinical severity scores were absent.

## Author contributions

PF analyzed the data and drafted the manuscript. SM, RA and EW collected the data and revised the manuscript. JL and EK designed the study, and partook in interpretation of data and revision of the manuscript. PFr interpreted the data and revised the manuscript. All authors discussed the results and commented on the manuscript.

## Funding

This work was supported by a grant from AbbVie, European Union Seventh Framework Programme (FP7/2007–2013) under grant agreement no 602919, Swedish Foundation for Strategic Research, Swedish Rheumatism Association, Swedish Research Council, Marianne and Marcus Wallenberg Foundation and the Stockholm County Council.

### Conflict of interest statement

The authors declare that the research was conducted in the absence of any commercial or financial relationships that could be construed as a potential conflict of interest.

## Supplementary material

Supplementary Table S1 **Individual usage of medication**. DMARD, Disease-modifying antirheumatic drugs.

Supplementary Table S2 **The table shows MNI coordinates and anatomical labels of the 159 seed regions used in the seed correlation analyses**. Anatomical labeling is performed using the automatic anatomical labeling (AAL) template in MRIcron. Stars (^*^) indicate absent AAL labels, in which case anatomical labeling was performed by using the Harvard-Oxford Cortical/Subcortical structural atlas or the Juelich Histological Atlas as provided by FSLview.

Supplementary Figure S1 **Spatial distribution of seed regions placed in pain processing brain regions**.
