# Supplementary material for: Intrinsic Brain Connectivity in Chronic Pain: A Resting-State fMRI Study in Patients with Rheumatoid Arthritis
Source: Front Hum Neurosci. 2016 Mar 15;10:107. doi: 10.3389/fnhum.2016.00107 (PMC4791375; doi:10.3389/fnhum.2016.00107)
Supplement: Supplementary Table S1 — Individual usage of medication. DMARD, Disease-modifying antirheumatic drugs. [file Table1.DOCX]

|  | **Steroids** | **Steroids, dosage/** | **DMARD** | **DMARD,**  dosage |
| --- | --- | --- | --- | --- |
|  |  | **day** |  |  |
| Subj 1 | No | N/A | Sulphasalazine | 500mgx2 |
| Subj 2 | No | N/A | Methotrexate | 25mg once weekly |
| Subj 3 | No | N/A | Methotrexate | 20mg once weekly |
| Subj 4 | Prednisolone | 5 mg | Methotrexate | 20mg once weekly |
| Subj 5 | No | N/A | Leflunomide | 10mgx1 |
| Subj 6 | No | N/A | Methotrexate | 20mg once weekly |
| Subj 7 | Prednisolon | 7,5 mg | Methotrexate | 20mg once weekly |
| Subj 8 | Prednisolon | 7,5mg | Methotrexate | 25mg once weekly |
| Subj 9 | Prednisolon | 3,75mg | No | N/A |
| Subj 10 | Prednisolon | 4,35mg | Methotrexate | 20mg once weekly |
| Subj 11 | No | N/A | Methotrexate | 15mg once weekly |
| Subj 12 | Prednisolon | 7,5mg | No | N/A |
| Subj 13 | Prednisolon | 7,5mg | Methotrexate | 20mg once weekly |
| Subj 14 | Prednisolon | 10mg | Methotrexate | 25mg once weekly |
| Subj 15 | Prednisolon | 7,5mg | Methotrexate & sulphasalazine | 20mg once weekly / 1000mgx1 |
| Subj 16 | Prednisolon | 7,5mg | Methotrexate | 20mg once weekly |
| Subj 17 | No | N/A | Methotrexate | 15mg once weekly |
| Subj 18 | Prednisolon | 5mg | Sulphasalazine & leflunomide | 1000mgx1 / 10 mgx1 |
| Subj 19 | Prednisolon | 7,5mg | Methotrexate | 20mg once weekly |
| Subj 20 | No | N/A | Sulphasalazine | 500mgx1 |
| Subj 21 | No | N/A | Methotrexate | 15mg once weekly |
| Subj 22 | Prednisolon | 7,5mg | Methotrexate | 20mg once weekly |
| Subj 23 | No | N/A | Methotrexate | 20mg once weekly |
| Subj 24 | Prednisolon | 7,5 mg | Methotrexate & sulphasalazine | 20mg weekly / 1000mgx2 |
