# Supplementary material for: Intrinsic Brain Connectivity in Chronic Pain: A Resting-State fMRI Study in Patients with Rheumatoid Arthritis
Source: Front Hum Neurosci. 2016 Mar 15;10:107. doi: 10.3389/fnhum.2016.00107 (PMC4791375; doi:10.3389/fnhum.2016.00107)
Supplement: Supplementary Table S2 — The table shows MNI coordinates and anatomical labels of the 159 seed regions used in the seed correlation analyses. Anatomical labeling is performed using the automatic anatomical labeling (AAL) template in MRIcron. Stars (*) indicate absent AAL labels, in which case anatomical labeling was performed by using the Harvard-Oxford Cortical/Subcortical structural atlas or the Juelich Histological Atlas as provided by FSLview. [file Table2.DOCX]

| **x** | **y** | **z** | **Region** |
| --- | --- | --- | --- |
| -30 | -6 | -12 | Amygdala |
| -10 | 24 | 28 | Anterior Cingulate Cortex |
| 0 | 14 | 28 | Anterior Cingulate Cortex |
| 0 | 24 | 28 | Anterior Cingulate Cortex |
| 0 | 34 | -2 | Anterior Cingulate Cortex |
| 0 | 34 | 18 | Anterior Cingulate Cortex |
| 0 | 34 | 28 | Anterior Cingulate Cortex |
| 10 | 24 | 28 | Anterior Cingulate Cortex |
| 10 | 34 | 28 | Anterior Cingulate Cortex |
| 10 | 44 | 8 | Anterior Cingulate Cortex |
| 0 | 24 | 18 | * Anterior Cingulate Cortex |
| 0 | -26 | -12 | * Brain stem |
| 0 | -26 | -22 | * Brain stem |
| 10 | -16 | -12 | * Brainstem |
| 10 | -26 | -12 | * Brainstem |
| 10 | 4 | 8 | * Caudate |
| 50 | 14 | 8 | Frontal Inferior operculum |
| 50 | 34 | -2 | Frontal Inferior operculum |
| 40 | 24 | 8 | Frontal Inferior Triangular |
| 40 | 34 | 28 | Frontal Inferior Triangular |
| 50 | 24 | 28 | Frontal Inferior Triangular |
| 50 | 24 | 8 | Frontal Inferior Triangular |
| 40 | 44 | 18 | Frontal Middle |
| -40 | -16 | 8 | Heschl's area |
| 50 | -16 | 8 | Heschl's area |
| 50 | -6 | 8 | Heschl's area |
| -20 | -6 | -12 | Hippocampus |
| -20 | -6 | -22 | Hippocampus |
| 20 | -6 | -12 | Hippocampus |
| 30 | -6 | -22 | Hippocampus |
| -40 | 24 | -2 | Inferior Frontal Gyrus Orbitalis |
| -50 | 14 | 8 | Inferior Frontal Gyrus Orbitalis |
| 0 | 54 | -12 | Inferior Frontal Gyrus Orbitalis |
| 40 | 14 | 8 | Inferior Frontal Gyrus Orbitalis |
| 40 | 24 | -12 | Inferior Frontal Gyrus Orbitalis |
| -40 | -36 | 48 | Inferior Parietal Lobe |
| -40 | -46 | 48 | Inferior Parietal Lobe |
| 50 | -36 | 48 | Inferior Parietal Lobe |
| 50 | -46 | 48 | Inferior Parietal Lobe |
| -30 | 24 | -2 | Insula |
| -30 | 24 | 8 | Insula |
| -40 | -16 | -2 | Insula |
| -40 | -6 | -2 | Insula |
| -40 | -6 | 8 | Insula |
| -40 | 14 | -12 | Insula |
| -40 | 14 | -2 | Insula |
| -40 | 14 | 8 | Insula |
| -40 | 4 | -2 | Insula |
| -40 | 4 | 8 | Insula |
| 30 | 24 | -2 | Insula |
| 30 | 24 | 8 | Insula |
| 40 | -16 | -2 | Insula |
| 40 | -16 | 8 | Insula |
| 40 | -6 | -2 | Insula |
| 40 | -6 | 8 | Insula |
| 40 | 14 | -2 | Insula |
| 40 | 24 | -2 | Insula |
| 40 | 4 | -12 | Insula |
| 40 | 4 | -2 | Insula |
| 40 | 4 | 8 | Insula |
| 50 | 4 | -2 | Insula |
| -30 | 14 | 8 | * Insula |
| 40 | 14 | -12 | * Insula |
| -10 | 14 | 38 | Middle Cingulate Cortex |
| -10 | 4 | 38 | Middle Cingulate Cortex |
| 0 | -26 | 28 | Middle Cingulate Cortex |
| 0 | -36 | 48 | Middle Cingulate Cortex |
| 0 | -6 | 38 | Middle Cingulate Cortex |
| 0 | -6 | 48 | Middle Cingulate Cortex |
| 0 | 14 | 38 | Middle Cingulate Cortex |
| 0 | 24 | 38 | Middle Cingulate Cortex |
| 0 | 4 | 38 | Middle Cingulate Cortex |
| 10 | -6 | 38 | Middle Cingulate Cortex |
| 10 | 14 | 38 | Middle Cingulate Cortex |
| 10 | 24 | 38 | Middle Cingulate Cortex |
| 10 | 4 | 38 | Middle Cingulate Cortex |
| -10 | 4 | -2 | Pallidum |
| -20 | 4 | -2 | Pallidum |
| 20 | 4 | -2 | Pallidum |
| 20 | -6 | -22 | ParaHippocampus |
| -30 | -36 | 58 | Postcentral |
| -40 | -26 | 48 | Postcentral |
| -40 | -26 | 58 | Postcentral |
| -50 | -16 | 18 | Postcentral |
| -60 | -16 | 18 | Postcentral |
| -60 | -16 | 28 | Postcentral |
| -60 | -6 | 18 | Postcentral |
| 0 | -36 | 28 | Posterior Cingulate Cortex |
| 0 | -46 | 28 | Posterior Cingulate Cortex |
| -50 | 4 | 28 | Precentral |
| 50 | 4 | 28 | Precentral |
| 50 | 4 | 38 | Precentral |
| -20 | 14 | -2 | Putamen |
| -20 | 4 | 8 | Putamen |
| 20 | 14 | -2 | Putamen |
| 20 | 14 | 8 | Putamen |
| 30 | 14 | -2 | Putamen |
| 30 | 14 | 8 | Putamen |
| 30 | 4 | -2 | Putamen |
| 30 | 4 | 8 | Putamen |
| -20 | 4 | -12 | * Putamen |
| -30 | 14 | -2 | * Putamen |
| -40 | -16 | 18 | Rolandic Operculum |
| -40 | -26 | 18 | Rolandic Operculum |
| -50 | -6 | 8 | Rolandic Operculum |
| -50 | 4 | 8 | Rolandic Operculum |
| 40 | -16 | 18 | Rolandic Operculum |
| 50 | -16 | 18 | Rolandic Operculum |
| 50 | 4 | 8 | Rolandic Operculum |
| 60 | -16 | 18 | Rolandic Operculum |
| 60 | 4 | 8 | Rolandic Operculum |
| 50 | 14 | -2 | * Rolandic Operculum |
| -10 | 4 | 48 | Supplementary Motor Areas |
| 0 | 14 | 48 | Supplementary Motor Areas |
| 0 | 14 | 58 | Supplementary Motor Areas |
| 0 | 24 | 48 | Supplementary Motor Areas |
| 0 | 4 | 48 | Supplementary Motor Areas |
| 10 | -6 | 48 | Supplementary Motor Areas |
| 10 | 14 | 48 | Supplementary Motor Areas |
| 10 | 14 | 58 | Supplementary Motor Areas |
| 10 | 24 | 48 | Supplementary Motor Areas |
| 10 | 4 | 48 | Supplementary Motor Areas |
| 10 | 4 | 58 | Supplementary Motor Areas |
| -50 | -26 | 28 | Supramarginal Gyrus |
| -50 | -36 | 28 | Supramarginal Gyrus |
| -60 | -26 | 28 | Supramarginal Gyrus |
| -60 | -26 | 38 | Supramarginal Gyrus |
| -60 | -36 | 28 | Supramarginal Gyrus |
| 50 | -26 | 28 | Supramarginal Gyrus |
| 50 | -36 | 28 | Supramarginal Gyrus |
| 60 | -16 | 28 | Supramarginal Gyrus |
| 60 | -26 | 28 | Supramarginal Gyrus |
| 60 | -26 | 38 | Supramarginal Gyrus |
| 60 | -36 | 28 | Supramarginal Gyrus |
| -50 | -26 | 18 | Temporal superior |
| -50 | 14 | -2 | Temporal superior |
| -50 | 4 | -2 | Temporal superior |
| -60 | -26 | 18 | Temporal superior |
| 50 | -26 | 18 | Temporal superior |
| 60 | -26 | 18 | Temporal Superior Gyrus |
| -10 | -16 | -2 | Thalamus |
| -10 | -16 | 8 | Thalamus |
| -10 | -26 | -12 | Thalamus |
| -10 | -26 | -2 | Thalamus |
| -10 | -26 | 8 | Thalamus |
| -10 | -6 | 8 | Thalamus |
| 10 | -16 | 8 | Thalamus |
| 10 | -6 | 8 | Thalamus |
| 20 | -16 | 8 | Thalamus |
| 0 | -16 | 8 | * Thalamus |
| 10 | -16 | -2 | * Thalamus |
| -20 | -6 | 8 | * White Matter |
| 0 | -16 | -2 | * White Matter |
| 0 | 4 | -2 | * White Matter |
| 10 | 4 | -2 | * White Matter |
| 20 | -6 | 8 | * White Matter |
| 40 | 4 | 18 | * White Matter |
| 10 | -6 | -2 | *White Matter |
| 10 | 14 | 28 | *White Matter |

*Supplementary table 1.* The table shows MNI coordinates and anatomical labels of the 159 seed regions used in the seed correlation analyses. Anatomical labeling is performed using the automatic anatomical labeling (AAL) template in MRIcron. Stars (*) indicate absent AAL labels, in which case anatomical labeling was performed by using the Harvard-Oxford Cortical / Subcortical tructural atlas or the Juelich Histological Atlas as provided by FSLview.
