# Supplementary figures and images for: Intrinsic Brain Connectivity in Chronic Pain: A Resting-State fMRI Study in Patients with Rheumatoid Arthritis
Source: Front Hum Neurosci. 2016 Mar 15;10:107. doi: 10.3389/fnhum.2016.00107 (PMC4791375; doi:10.3389/fnhum.2016.00107)

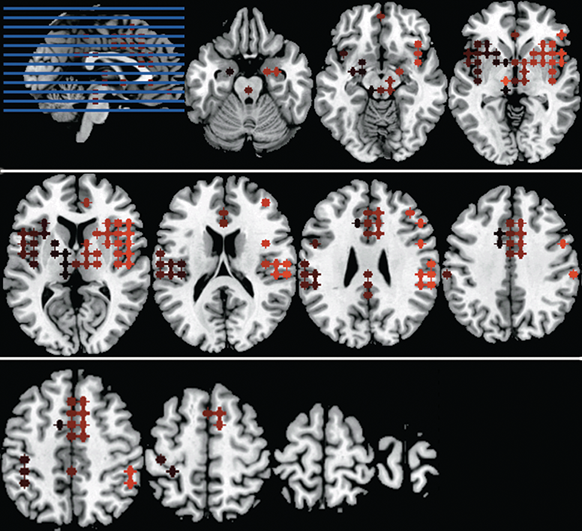

Supplement: Supplementary Figure S1 — Spatial distribution of seed regions placed in pain processing brain regions. [file Image1.TIF]
